# Topochemical Synthesis and Electronic Structure of High-Crystallinity Infinite-Layer Nickelates on an Orthorhombic Substrate

**DOI:** 10.1021/acs.nanolett.4c06557

**Published:** 2025-01-09

**Authors:** Zhengang Dong, Marios Hadjimichael, Bernat Mundet, Jaewon Choi, Charles C. Tam, Mirian Garcia-Fernandez, Stefano Agrestini, Claribel Domínguez, Regan Bhatta, Yue Yu, Yufeng Liang, Zhenping Wu, Jean-Marc Triscone, Chunjing Jia, Ke-Jin Zhou, Danfeng Li

**Affiliations:** † State Key Laboratory of Information Photonics and Optical Communications, School of Science, Beijing University of Posts and Telecommunications, Beijing 100876, China; ‡ Department of Physics, Hong Kong Institute for Advanced Study, City University of Hong Kong, Kowloon, Hong Kong 999077, China; § City University of Hong Kong Shenzhen Research Institute, Shenzhen, Guangdong 518057, China; ∥ Department of Quantum Matter Physics, University of Geneva, 24 Quai Ernest-Ansermet, 1211 Geneva, Switzerland; ⊥ Department of Physics, University of Warwick, Coventry, CV4 7AL, United Kingdom; # Electron Spectrometry and Microscopy Laboratory (LSME), Institute of Physics (IPHYS), École Polytechnique Fédérale de Lausanne (EPFL), 1015 Lausanne, Switzerland; ∞ Catalan Institute of Nanoscience and Nanotechnology (ICN2), Campus UAB, Bellaterra, Barcelona, 08193, Catalonia, Spain; ○ Diamond Light Source, Harwell Campus, Didcot OX11 0DE, United Kingdom; $ H.H. Wills Physics Laboratory, University of Bristol, Bristol BS8 1TL, United Kingdom; % Department of Physics, University of Florida, Gainesville, Florida 32611, United States; & Department of Computer Science, University of Florida, Gainesville, Florida 32611, United States; @ The Molecular Foundry, Lawrence Berkeley National Laboratory, Berkeley, California 94720, United States

**Keywords:** infinite-layer nickelates, topochemistry, NaH, orthorhombic substrates, off-axis RF magnetron sputtering, electronic structure

## Abstract

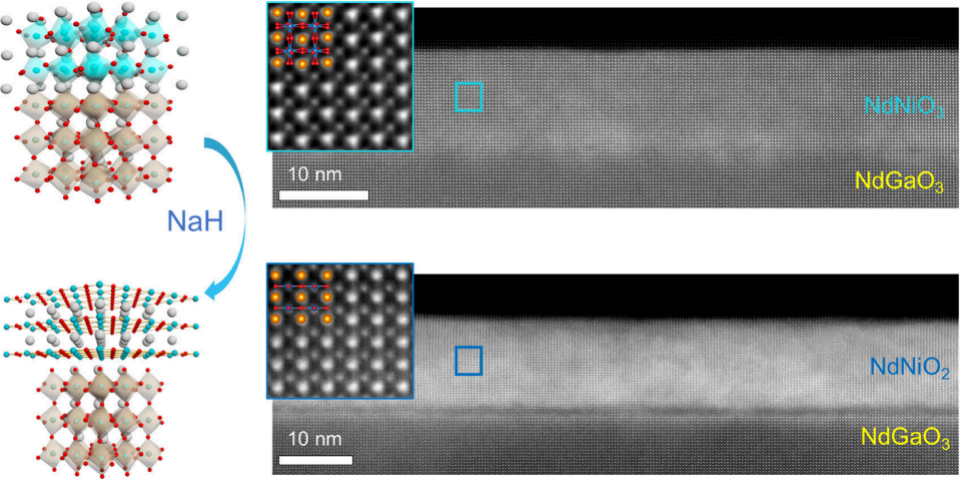

Superconductivity in infinite-layer nickelates has stirred much research interest, to which questions regarding the nature of superconductivity remain elusive. A critical leap forward to address these intricate questions is through the growth of high-crystallinity infinite-layer nickelates, including the “parent” phase. Here, we report the synthesis of a high-quality thin-film nickelate, NdNiO_2_. This is achieved through the growth of a perovskite precursor phase (NdNiO_3_) of superior crystallinity on the NdGaO_3_ substrate by off-axis RF magnetron sputtering and a low-temperature topochemical reduction using NaH. We observe a nonlinear Hall effect at low temperatures in this “non-doped” phase. We further study the electronic properties using advanced X-ray scattering and first-principles calculations. We observe spectroscopic indications of the enhanced two-dimensionality and a reduced hybridization of Nd 5*d* and Ni 3*d* orbitals. These findings unlock new pathways for preparing high-quality infinite-layer nickelates and provide new insights into the intrinsic features of these compounds.

Nickelates have emerged as a new class of high-temperature superconductors (HTSs) possessing various lattice structures.^1−5^ Among these compounds, infinite-layer nickelates with 3*d*^9^ electronic configuration host unconventional superconductivity^1^ with intriguing properties that are both analogous and distinct to that of high *T*_c_ (*T*_c_, superconducting transition temperature) cuprates.^6^ Driven by this notion, immediate fundamental questions for this materials system regarding electronic structure, gap symmetry, effects of multiple orbitals, pairing interaction, etc., have been the focus of many theoretical considerations.^7−11^ However, largely owing to the constraints on sample volume/geometry and the challenges in materials growth, experimental approaches to these enigmas are often limited.^12−25^ This is possibly due to the variations in materials growth conditions and the existence of closely related competing thermodynamic phases.^26^

Despite this, continuing efforts in materials synthesis aiming at suppressing structural disorder have enabled pivotal findings in thin films with improved quality.^23,27^ What remains less understood and intriguingly distinct from the properties of cuprates is the lack of a clear Mott-type insulating state^1,27−30^ and the possible presence of a proximate correlated charge-stripe phase^20−22^ even at the nominal zero doping. These generic observations have framed much of the ongoing debate about the nature of the “undoped” parent phase from which superconductivity emerges. On the materials side, reducing the density of both dopants and defects inevitably lessens the kinetics of ionic transport during topochemical reduction, making it difficult to reach uniform and attainable infinite-layer phases.^27^ To this end, one central research task is to prepare and investigate high-quality non-doped parent compounds. Here, we synthesize infinite-layer nickelate thin films from a NdNiO_3_ (NNO3) precursor deposited using off-axis radiofrequency (RF) magnetron sputtering possessing a very high crystallinity. Instead of the widely used calcium hydride (CaH_2_),^1,31^ we employ the more reactive sodium hydride (NaH) as the reducing reagent^32,33^ and take full advantage of its intensive reductive power at lower temperatures,^34^ which promotes the topotactic oxygen de-intercalation while retaining the lattice stability. Transport measurements reveal resistivity drops at low temperatures and nonlinear Hall resistivity in the normal state in these high-quality samples. We further report spectroscopic features and electronic band configurations that show clear differences and may originate from the orthorhombicity of the substrates.

The NNO3 films, which were subsequently annealed to NdNiO_2_ (NNO2) phases through topochemical reduction, were grown on (110)_o_-oriented NdGaO_3_ (NGO; the subscript “o” denotes “orthorhombic”; equivalent to the (001) pseudocubic (pc) direction, which is used for convenience in this article) substrates by off-axis RF magnetron sputtering (see the Supporting Information). This technique has been widely used to produce remarkably high-quality nickelate thin films.^35,36^

Figures 1a and 1b show the schematic crystal structures of the pristine NNO3 films and infinite-layer NNO2 films on NGO substrates, respectively. The bulk precursor NdNiO_3_ has an orthorhombic structure (space group *Pbnm*) with *a*_0_ = 5.389 Å, *b*_0_ = 5.382 Å, and *c*_0_ = 7.610 Å. NdGaO_3_ also possesses an orthorhombic structure (space group *Pbnm*), with lattice parameters of *a*_0_ = 5.433 Å, *b*_0_ = 5.503 Å, and *c*_0_ = 7.716 Å. For NNO3 films grown on NGO (001)_pc_ substrates, the *in-plane* lattice mismatches are 1.37% along [100] and 1.51% along [010]. As shown in Figure 1d, the X-ray diffraction (XRD) scan of an NNO3 film on the (001)_pc_ NGO substrate reveals a high-quality pseudocubic perovskite phase marked by sharp 00*l* peaks with high peak intensity and well-defined Laue finite-size oscillations.

**Figure 1:**
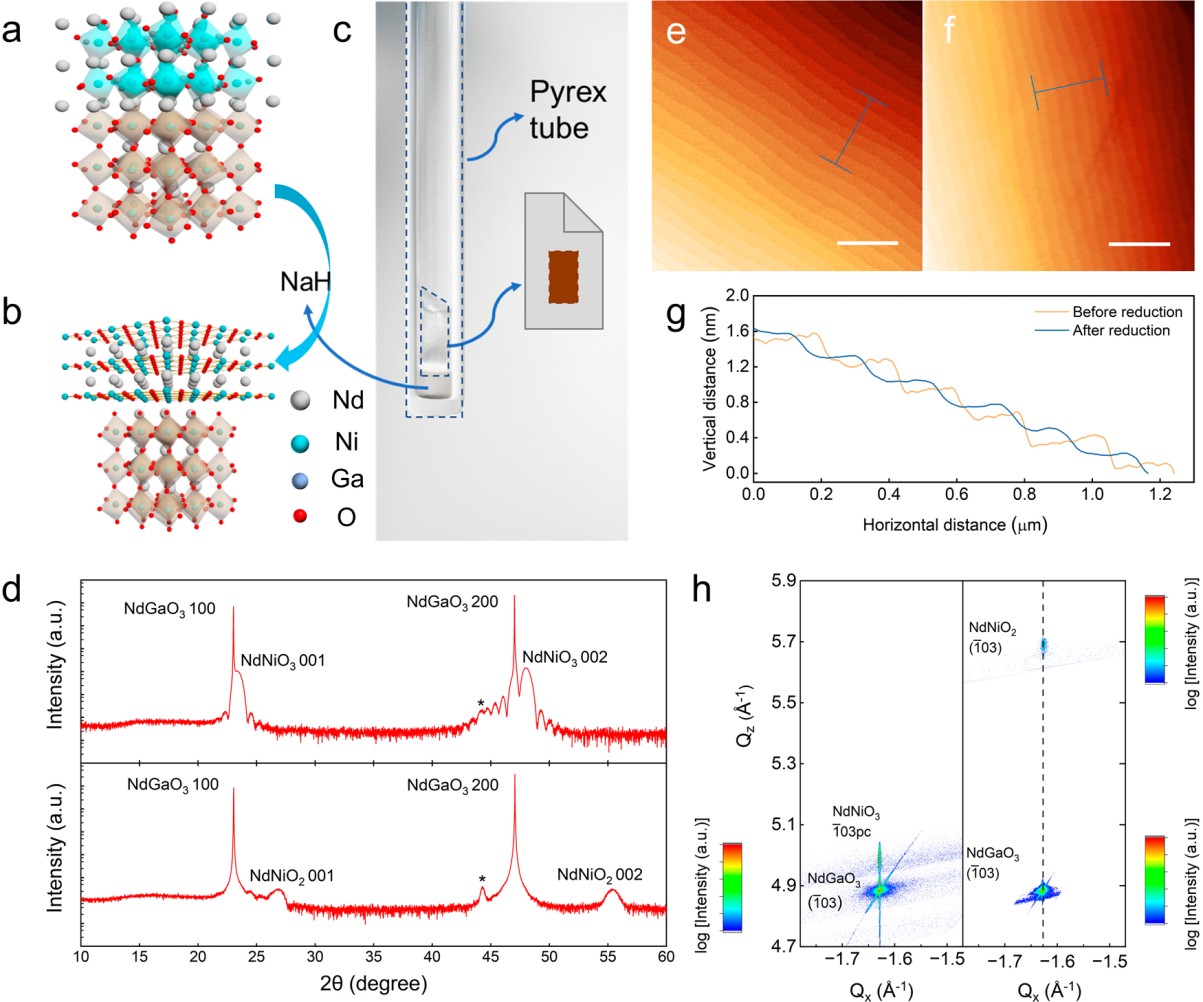
**Structural characterization of NdNiO_3_ and NdNiO_2_ films on (001)_pc_-oriented NdGaO_3_ substrates.** Schematic crystal structure of (a) NdNiO_3_ grown on NdGaO_3_ and (b) NdNiO_2_ on NdGaO_3_. After reduction using NaH, the structure changes from perovskite to the infinite-layer phase. (c) Optical image of the Pyrex tube with the sample wrapped in aluminum foil and NaH powder inside the tube. (d) Specular θ–2θ scans around the 001 and 002 peaks of the NdGaO_3_ substrate for the NdNiO_3_ film (top) and the NdNiO_2_ film (bottom). * denotes the unfiltered parasitic peak from the diffractometer. Upon topochemical reaction, the out-of-plane parameter changes from 3.787 to 3.315 Å. (e, f) AFM topography of thin films before and after reduction, respectively. The scale bars represent 1 μm. (g) The line profile of thin films before and after reduction. (h) Reciprocal space maps around the 1̅03 peak of the NdGaO_3_ substrate for the NdNiO_3_ film (left) and the NdNiO_2_ film (right). Each map was completed through multiple scans.

Our initial attempts to reduce the precursor phase using CaH_2_ failed, as the annealing process destabilized and decomposed the pristine perovskite phase. Figure S1 in the Supporting Information shows a representative reduction process with CaH_2_: for reduction temperature up to 340 °C (substantially higher than those in the literature^21,22,26,29,37−39^), the 002 film peaks in the symmetric θ–2θ scans show an almost negligible change. This may be related to the fewer defect sites due to higher crystallinity and absence of dopants, which potentially inhibit oxygen ionic transport. Upon further increasing the temperature to 360 °C for a prolonged dwelling time, the 002 peak disappears, signifying structural decomposition of the film. We attribute this decomposition to the structural instability of NNO3 promoted by the ionic transport at high temperatures. We, therefore, need a more aggressive reducing reagent that functions at lower active temperatures.

NaH is a clear choice for its massive H production at lower temperatures^34^ and has been broadly used in topochemical reduction reactions for various oxides.^32−34,40−42^ Using NaH, we successfully achieved the NNO2 phase with a *c*-axis of 3.31 Å. This is evidenced in XRD θ–2θ symmetric scans (Figure 1d and Figure S2) displaying two film peaks corroborating the formation of the [001]-oriented NNO2 phase. We note that the topotactic transition with the *c*-axis lattice parameter changing from 3.79 to 3.31 Å occurs at temperatures as low as ∼210 °C, remarkably lower than the destabilizing temperature when CaH_2_ is used. A series of NNO2 films were synthesized with the 2θ values of their 002 reflections and the calculated *c*-axis lattice parameters shown in Table S1 (see the Supporting Information). Figures 1e and 1f show the atomic force microscopy (AFM) topographic images of the NNO3 and NNO2 films. For both before and after reduction, the film surface retains atomically flat step-terrace morphology. To further investigate the in-plane epitaxial relationship and strain state, we performed the reciprocal space mapping (RSM) measurements around the 1̅03 peaks in pseudocubic notation. The data are illustrated in Figure 1h, in which both substrate and film peaks occur at the same values of Q_*x*_, indicative that the films are epitaxially strained to NGO substrates. As compared with the reported value for bulk NNO2 (∼3.92 Å), the film experiences an in-plane compressive strain of up to 1.53%.^33^

To further assess the quality and investigate the structure of the films, STEM experiments were performed. Figures 2a and 2c show the wide range high-resolution high-angle annular dark-field STEM (HAADF-STEM) images for the NNO3 and NNO2 thin films, while their respective fast Fourier transform (FFT) patterns are plotted in Figures 2b and 2d. The as-grown NNO3 phase exhibits excellent crystalline integrity across the investigated region of the film without traceable defects or stacking faults. The FFT image in Figure 2b shows a clear set of perovskite reflections for both NNO3 film and NGO substrate, as indicated by the horizontal arrows. These primary spots are also accompanied by strong half-order peaks that originate from octahedral tilt-induced distortions in both the film and the substrate. We note that the location of these half-order peaks is the same, corroborating that the NNO3 film nucleates with the same lattice orientation as that of the NGO substrate everywhere. We believe that this monodomain configuration has a remarkable influence on the observed suppression of lattice defects in the film.^36^ The high-quality epitaxy is further evidenced by the sharp interface between the NNO3 film and the NGO substrate, as observed in the high-magnification image (Figure 2e).

**Figure 2:**
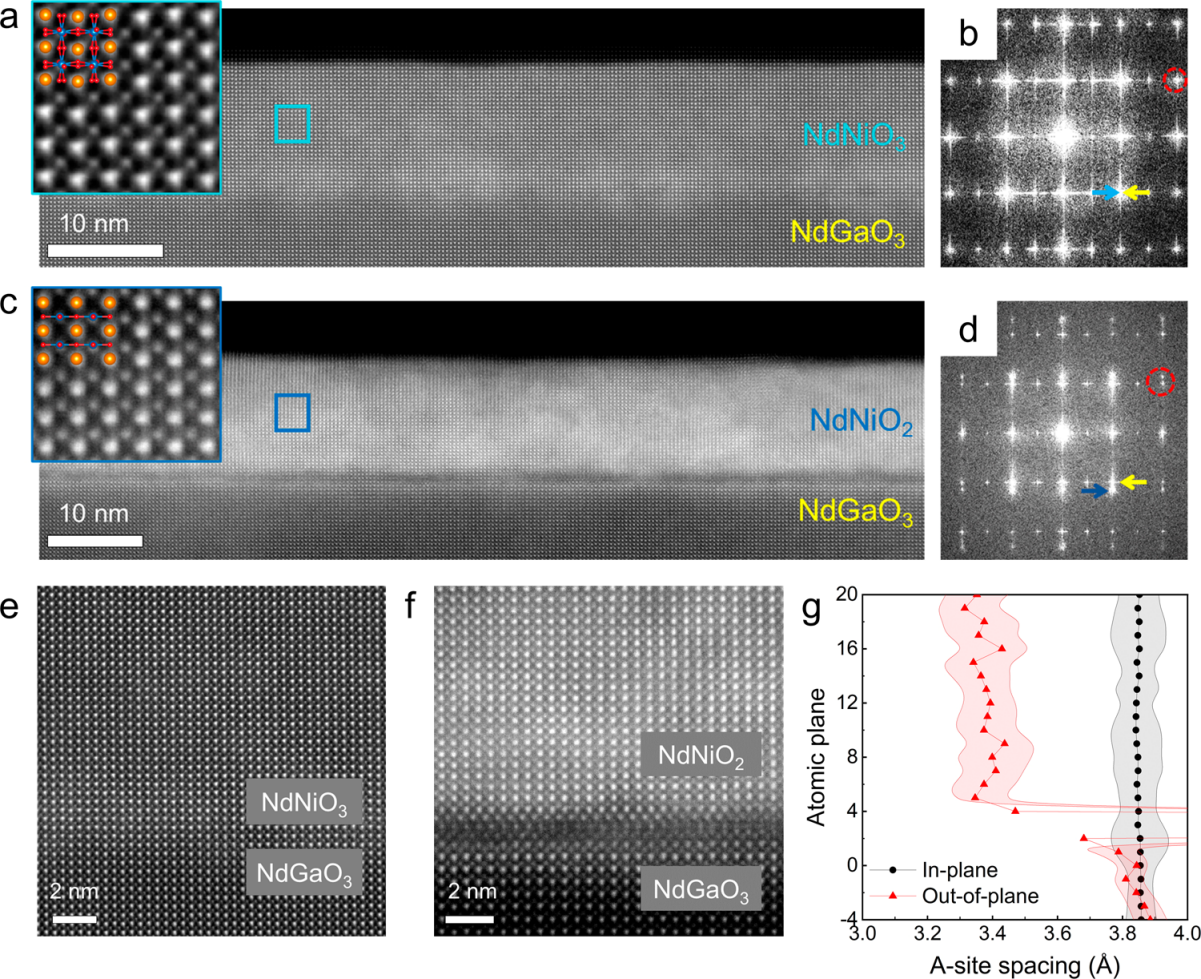
**STEM images of pristine perovskite phase and infinite layer phase films.** (a, c) Large-scale atomic-resolution HAADF-STEM images of NdNiO_3_ and NdNiO_2_ films, respectively. Insets: the atomic configurations are illustrated by orange, blue, and red spheres for Nd, Ni, and O atoms, respectively. (b, d) The FFT patterns of the large-scale images, corresponding to the pristine and reduced samples, respectively. The arrows (with the same color code as in (a) and (c)) indicate the main reflections of the NdGaO_3_ substrate and the films. The different patterns marked by red circles in (b) and (d) illustrate the change in *c*-axis constants of the films in comparison to that of the NdGaO_3_ substrate. (e, f) The enlarged HAADF-STEM images of the interface regions with films of perovskite phase and infinite-layer phase, respectively. (g) A-site spacing as a function of the atomic plane across the interface extracted from (f) for NdNiO_2_/NdGaO_3_.

On the other hand, even though the film/substrate is perfectly coherent for the NNO2 case as well, a thin interfacial layer extending 2–3 u.c. (giving rise to the darker contrast) is observed at the interface, as shown in Figure 2c. We argue that this planar layer is present for two reasons: to accommodate the strain pertaining to the substrate surface step edges due to the large difference in the *c*-axis lattice constant of NNO2 and NGO and to connect the two incommensurable octahedral tilt patterns associated with the *Pbnm* NGO structure (a^–^a^–^c^+^) and the infinite-layer NNO2 lattice. The FFT of Figure 2c, shown in Figure 2d, clearly illustrates that the reflections associated with NNO2 now occur at a different vertical position (marked by a blue arrow; versus a yellow arrow for substrate), showing the large change in *out-of-plane* lattice parameter. In agreement with our XRD measurements, the in-plane lattice parameter remains the same, as evidenced by the horizontal alignment of the NGO and NNO2 reflections. We note that the half-order peaks associated with the NGO and NNO3 structures do not exist for NNO2. We also note that no additional peaks associated with oxygen non-stoichiometry and incomplete reduction^43^ have been observed.

Upon reduction by NaH,^32−34^ a uniform infinite-layer phase forms, as illustrated in Figure 2c and also in Figure 2f for a partially enlarged view. The shrinkage of the out-of-plane lattice constant upon topotactic phase transitions can also be directly quantified using the coordinates of the atomic columns of the A-site sublattice in Figure 2f. As a result, the lattice parameters are plotted as depth profiles in Figure 2g. The in-plane parameter remains constant across the interface, in line with the fact that the films are strained to the substrate (Figure 1g). A sudden drop in the out-of-plane parameter is identified for the infinite-layer structure. The obtained *c*-axis parameter averaged across the thin film (∼3.33 Å) is close to the value extracted from the XRD data.

We then turn to investigate the transport properties of these films. The details of making electrical contacts for the measurements can be found in the Supporting Information. Figure 3a shows the temperature-dependent resistivity for as-grown NNO3 films from room temperature to 2 K. A sharp first-order metal–insulator transition (MIT) can be revealed in the resistivity data, with a transition temperature (*T*_MI_) of ∼160 K, which is consistent with the previously reported values for NNO3 films on NGO substrates.^35,44−46^Figure 3b exhibits the resistivity data as a function of the temperature for multiple NNO2 films. The resistivity values of all of the NNO2 samples investigated can be found in Table S1 (see the Supporting Information). We can make the following observations. First, despite scattered room-temperature resistivity values among samples, a metallic behavior down to low temperatures can generally be obtained, in line with the literature reports. Next, for most samples, an upturn in resistivity is observed, consistent with previous studies on NNO2 films.^27−29^ Last, we note that, for a few samples, a small dip in resistivity at the lowest temperature is present (Figure 3b; the low-temperature resistivity of a narrow temperature range, its magnetic-field dependence, and the discussion on their possible origin are shown in the Supporting Information).

**Figure 3:**
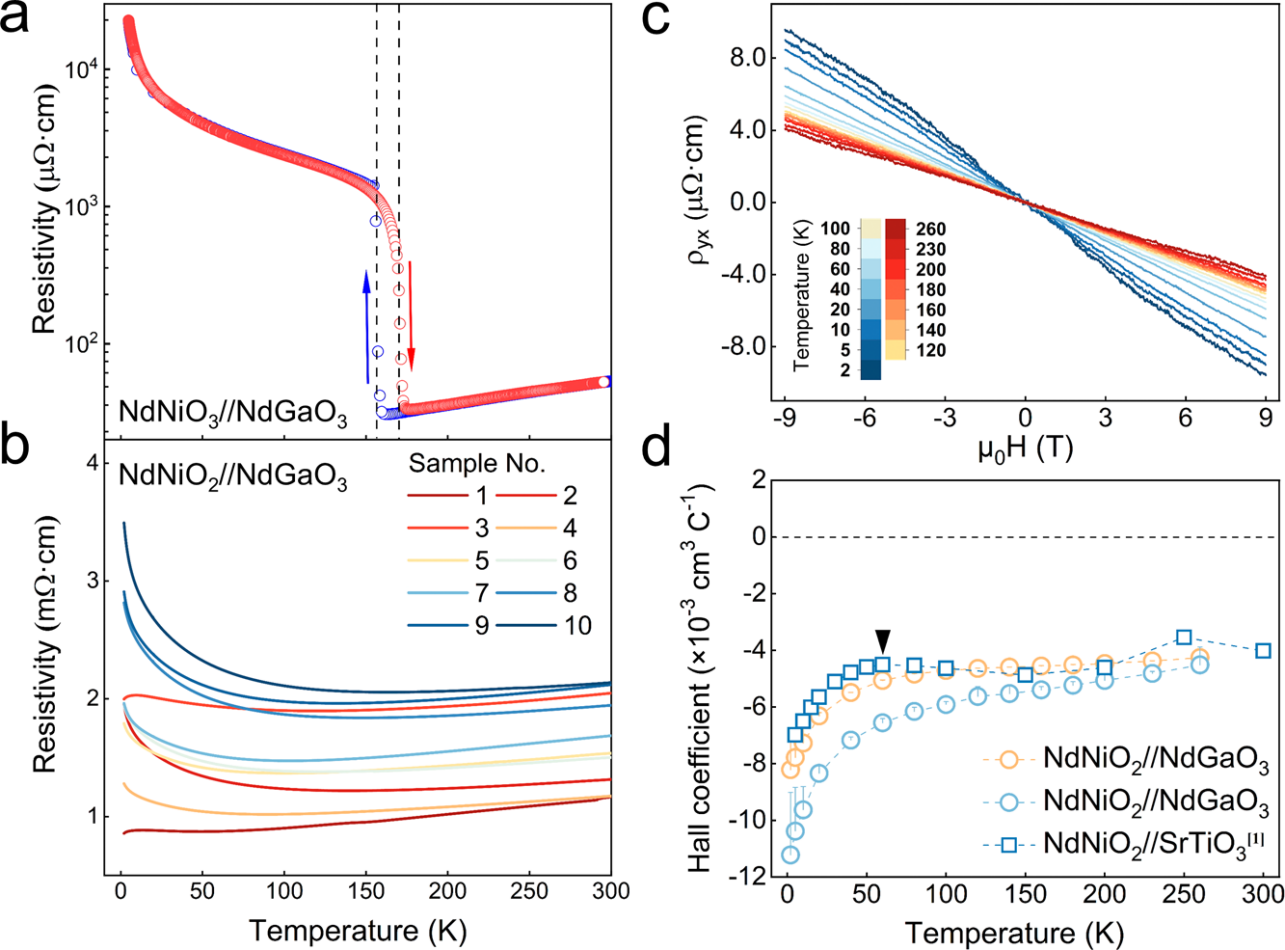
**Transport properties of the pristine and reduced films.** (a, b) Resistivity versus temperature curves of a representative NdNiO_3_ film and multiple NdNiO_2_ films on the NdGaO_3_ substrate. The data set for the NdNiO_3_ film shows a clear MIT transition. Most data for NdNiO_2_ films show a metallic behavior down to low temperatures before a resistivity upturn occurs. Two samples display a clear drop in resistivity as the temperature is lowered toward base temperature (2 K). (c) Hall resistivity (ρ_*yx*_) at various temperatures of Sample 4 shows a nonlinear Hall effect at low temperatures. (d) The temperature dependence of the normal-state Hall coefficients (fitted from 9 to −9 T and from 7 to 9 T as the error bar), in comparison to the literature data of NdNiO_2_ on the SrTiO_3_ substrate. Adapted with permission from ref (1). Copyright 2019 Springer Nature. The black arrow indicates the local minimum in the Hall coefficient value as a function of temperature for the NdNiO_2_/SrTiO_3_ data set. This minimum is not present for the NdNiO_2_/NdGaO_3_ films.

By using transverse electrical contacts, we examined the Hall signals of the NNO2 films. To avoid the influence of the possible superconducting fluctuation, we chose the sample with low resistivity but no resistivity downturn (Sample 4) and measured the Hall resistivity (ρ_*yx*_) across the entire temperature range (from 260 to 2 K). The data are shown in Figure 3c. Apart from a consistent negative slope in ρ_*yx*_(*H*) revealing electrons as the majority of the charge carriers,^27−29,47^ a nontrivial nonlinear field dependence is observed. This clear nonlinearity feature has never been reported in previous studies of NNO2 thin films on SrTiO_3_ (STO)^28^ and could be an electronic transport signature of the multipocket features of the Fermi surface of the undoped compound, which have recently been directly observed by angle-resolve photoemission spectroscopy thanks to improved crystallinity.^48,49^ Additionally, we extract the Hall coefficient of the measured two samples by approximate linear fitting at a wide range of temperatures and compare them with the reported NNO2 on STO substrates.^1^ They show similar trends apart from the fact that no local minimum in Hall coefficient value at low temperatures (indicated by the black triangle in the NNO2/STO data set) is present.

We performed X-ray absorption spectroscopy (XAS) and resonant inelastic X-ray scattering (RIXS) measurements at the Ni *L*-edge to experimentally access the electronic structure of the NNO2 thin films. Figure 4a shows the fluorescence-yield XAS spectra, which reflect the bulk averaged information. Two resonance peaks at ∼852 and ∼869 eV can be assigned to the Ni *L*_3_ and *L*_2_ absorption edges, respectively. Due to the nature of the nominal *d*^9^ states of NNO2, *I*_*ab*_ (*I*_*c*_) is proportional to the unoccupied density of states (DOS) with the *d*_*x*^2^–*y*^2^_ (*d*_*z*^2^_) orbital character. The single-peak profile of *I*_*ab*_*L*_3_-XAS is consistent with that of LaNiO_2_ and NNO2 films grown on (and capped with) STO.^15,20^ The large anisotropy, namely, more predominant *L*_3_ along *I*_*ab*_ than *I*_*c*_, reflects that most holes reside in the *d*_*x*^2^–*y*^2^_ orbital. In Figure 4c,d, we present the incident-energy dependent RIXS maps collected with σ and π polarized X-rays at Q = (−0.35, 0). For the applied experimental geometry, the wave vector (−0.35, 0) corresponds to a grazing incident angle of 35°. In this case, the energy-dependent RIXS spectra collected using π-incident polarization (Figure 4c) contain a major component along the *c*-axis. The low energy region (from 0 to 300 meV) comprises the quasi-elastic peak, phonon, and magnon. In the higher energy region (from 300 meV to 3 eV), dominant features are Ni *dd* excitations located at ∼1.4 and ∼2.0 eV. The signature of Nd 5*d*–Ni 3*d* hybridization at ∼0.6 eV is visible but much weaker than the *dd* excitations. The general features of the RIXS spectra are consistent with those of the parent NNO2 film grown on (and capped with) STO.^15,50^

**Figure 4:**
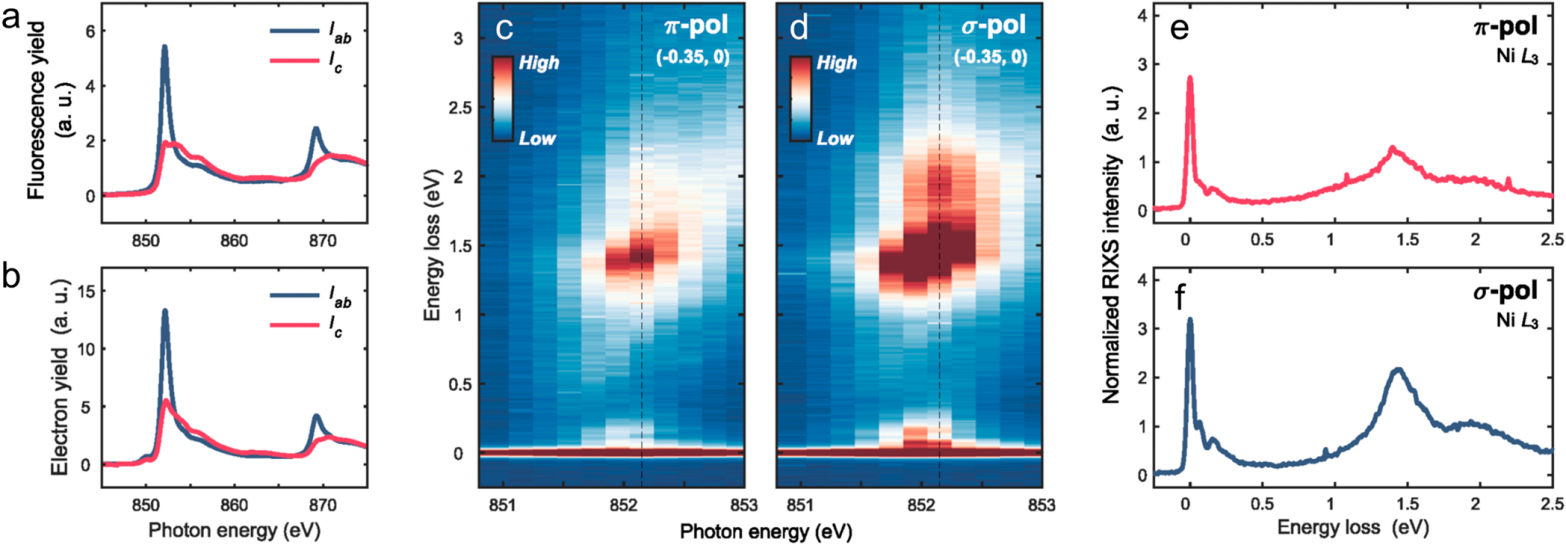
**Ni *L*-edge XAS and RIXS of the NdNiO_2_ film on the NdGaO_3_ substrate.** (a, b) Ni *L*-edge XAS spectra parallel to the *ab* plane (*I_ab_*) and the *c*-axis (*I*_*c*_) collected using the bulk-sensitive fluorescence yield (a) and the surface-sensitive electron yield (b). (c, d) RIXS maps with incident photon energy varied across the Ni *L*_3_ edge on NdNiO_2_ film at 20 K at *Q* = (−0.35, 0) r.l.u. with π (c) and σ (d) incident X-ray polarization. (e, f) Representative RIXS line spectra excited at the Ni *L*_3_ main peak ∼850.8 eV, indicated by dashed lines in (c) and (d). a.u. refers to arbitrary units.

To gain insights into the electronic structure of the strained NNO2 thin film, considering both its bulk properties and its interface with the NGO substrate, we performed density functional theory (DFT) calculations on strained bulk NNO2 as well as a superlattice composed of layered NNO2 on the NGO (001)_pc_ substrate, as shown in Figure 5. The structural relaxation and electronic structure calculations were carried out using the PBE functional^51,52^ implemented in Quantum Espresso.^53^ In Figure 5a, the DFT-calculated band structure for bulk NNO2 strained to the NGO substrate is compared to that for bulk NNO2 strained to the more commonly used STO substrate. The in-plane lattice parameters for NNO2 on the NGO (001)_pc_ substrate are approximately 1.2% smaller than those for NNO2 on the STO substrate. The calculated band structure displays a larger bandwidth for bulk NNO2 on the NGO (001)_pc_ substrate, consistent with the expectation that smaller in-plane lattice parameters result in increased hoppings and larger bandwidth, although it is not significant due to the small difference in strain imposed by different substrates.

**Figure 5:**
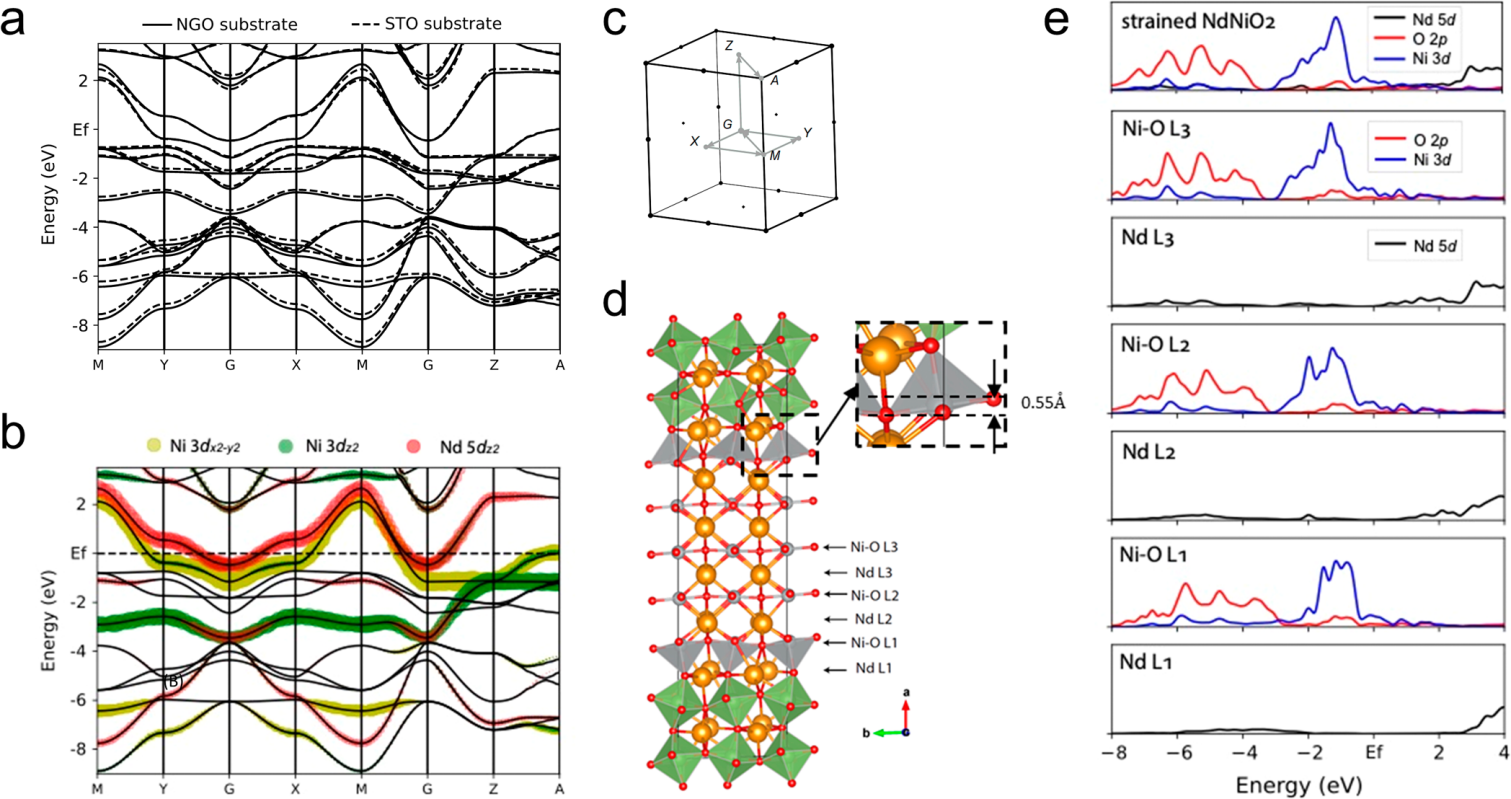
**Density functional theory calculations of the electronic structure for strained NdNiO_2_ near the interface with NdGaO_3_ substrate.** (a) DFT-calculated electronic band structure for bulk NdNiO_2_ in the paramagnetic state strained to NdGaO_3_ substrate (solid line) and SrTiO_3_ substrate (dashed line). (b) Orbitally resolved band structure of bulk NdNiO_2_ in the paramagnetic state strained to the NdGaO_3_ substrate, highlighting the orbital contents for Ni 3*d*_*x*^2^–*y*^2^_ (yellow), Nd 5*d*_*z*^2^_ (red), and Ni 3*d*_*z*^2^_ (green), respectively. (c) High symmetry momentum points and first Brillouin zone for paramagnetic bulk NdNiO_2_ strained to NdGaO_3_ substrate. (d) Heterostructure of layered NdNiO_2_ with NdGaO_3_ substrate, optimized through DFT calculations. The zoomed structure emphasizes the distorted in-plane oxygen at the interface. (e) Partial density of states for bulk NdNiO_2_ and cross different layers in the heterostructure, as noted in panel (d).

Next, we explore the structural distortion and corresponding electronic structure at the interface of NNO2/NGO. The details of the constructed superlattice and the DFT calculations can be found in the Supporting Information. The first Ni–O layer (Ni–O L1) adjacent to the NGO substrate, as depicted in Figure 5d in the relaxed structure, exhibits an oxygen distortion of approximately 0.55 Å along the lattice parameter direction [100], roughly half of the oxygen distortions in the alternating octahedra of NGO. As we move away from the substrate to the second and third layers (Ni–O L2 and Ni–O L3) of NNO2, this distortion rapidly diminishes.

Figure 5e illustrates the layer-dependent orbital-resolved partial density of states (PDOS), compared with the PDOS of bulk NNO2. The results show that the Ni 3*d* orbital in the Ni–O L1 layer at the interface displays a narrower distribution and the charge transfer energy for oxygen is lower, compared to those in Ni–O L2 and Ni–O L3. Although this feature is prominent for L1, the PDOS for L2 becomes closer to that of bulk NNO2, while the PDOS for L3 appears almost indistinguishable from that of the bulk NNO2. These indicate that the substrate’s influence on the electronic structure of NNO2 primarily affects the first two layers moving away from the substrate.

Just as much as on the nature of the superconducting phase, important aspects regarding the undoped phase remain unknown. For instance, at zero doping (*x* = 0 for Nd_1–*x*_Sr_*x*_NiO_2_), the presence of electron bands across the Fermi energy is associated with a “self-doping” mechanism in the NNO2 system.^11,30,54,55^ This feature is at the center of the multiband nature of the infinite-layer nickelate systems and brings about discussions on the role of rare-earth (i.e., Nd) 5*d* and other orbitals.^56,57^ Our work here allows us to revisit these issues through the transport and resonant X-ray scattering experiments.

Perhaps equally intriguing in our study are the NGO (001)_pc_ substrate and the impact that it may have on the resulting infinite-layer phase. Through epitaxy, a high-quality lattice-matched substrate enables the growth of precursor NNO3 films of high crystallinity and simultaneously offers the possibility to stabilize the NNO2 phase of different *a*- and *b*-lattice constants, due to the orthorhombic nature of the substrate.

Compared to that of the NNO2 film grown on the STO substrate,^50^ our Ni *L*_3_-XAS data present a relatively higher anisotropy (*i.e*., higher *I*_*ab*_/*I*_*c*_ ratio), alluding to a more two-dimensional electronic structure of NNO2/NGO. Interestingly, the excitation at ∼0.6 eV appears to show lower intensity reflecting potentially a weaker Ni 3*d*–Nd 5*d* hybridization. These differences may result from the fact that the system has a more two-dimensional electronic structure and a slightly different local crystal field owing to the strain imposed by the NGO substrate.

In summary, we have successfully produced infinite-layer NdNiO_2_ from high-quality NdNiO_3_ thin films on an orthorhombic substrate grown by low-cost off-axis RF magnetron sputtering (potentially beneficial for future applications). We have employed a powerful and reactive NaH and achieved high-quality undoped NNO2 thin films. In these films with zero doping, the nonlinear Hall resistivity was observed. X-ray spectroscopy and scattering measurements demonstrated a *d*^9^ electronic configuration with a large orbital polarization in the *d*_*x*^2^–*y*^2^_ orbital and a weakened hybridization between the Nd 5*d* orbitals and Ni 3*d* orbitals. Our DFT calculations on the pristine NNO2/NGO heterostructure mark sizable differences in the configuration of Ni 3*d* and O 2*p* orbitals at the interfaces, offering insights into the effect of a distorted perovskite structure on the electronic structure of infinite-layer nickelate thin films.

## Data Availability

All data needed to evaluate the conclusions in the paper are present in the paper and/or the Supporting Information. Raw data can be obtained from the corresponding authors upon reasonable request.

## Supporting Information Available

Additional materials and methods, experimental details of the thin-film growth and reduction processes and characterization methods, and experimental and calculation data, including growth of precursor films; reduction processes; X-ray diffraction and atomic force microscopy; scanning transmission electron microscopy; transport measurements; X-ray absorption spectroscopy and resonant inelastic X-ray scattering; density functional theory calculations; lattice parameters of infinite layer nickelate determined using STEM; visualization of oxygen atoms using annular bright field imaging; the strain distribution of infinite layer nickelate; XRD pattern of the reduction process using CaH_2_ and NaH; ABF-STEM image of the NdNiO_3_ film and the NdNiO_2_ film; the enlarged HADDF-STEM image and strain distribution of the interface region; magnetoresistance of representative samples; DFT calculated band structure; the experimental geometry of XAS and RIXS measurements; XRD data, fitting thickness, and resistivity of various NNO2 samples (PDF)

## Notes

During the peer review process of this work, we became aware of two preprints that report the observation of a superconducting state in NdNiO_2_ on STO and LSAT substrates^58^ and PrNiO_2_ on STO^59^ substrates, which may be in line with some of our findings here.
