# Supplementary material for: Topochemical Synthesis and Electronic Structure of High-Crystallinity Infinite-Layer Nickelates on an Orthorhombic Substrate
Source: Nano Lett. 2025 Jan 9;25(3):1233–41. doi: 10.1021/acs.nanolett.4c06557 (PMC11760177; doi:10.1021/acs.nanolett.4c06557)
Supplement: Supplementary file 1 — nl4c06557_si_001.pdf [file nl4c06557_si_001.pdf]

Supporting Information for

Topochemical synthesis and electronic structure of high-crystallinity infinite-layer nickelates on an orthorhombic substrate

Zhengang Dong[a, b, c§], Marios Hadjimichael[d, e§], Bernat Mundet[d, f, g], Jaewon Choi[h], Charles C. Tam[h, i], Mirian Garcia-Fernandez[h], Stefano Agrestini[h], Claribel Domínguez[d], Regan Bhatta[j], Yue Yu[k], Yufeng Liang[l], Zhenping Wu[a*], Jean-Marc Triscone[d], Chunjing Jia[j*], Ke-Jin Zhou[h*], Danfeng Li[b, c*]

[a]*State Key Laboratory of Information Photonics and Optical Communications, School of Science, Beijing University of Posts and Telecommunications, Beijing 100876, China*

[b]*Department of Physics, Hong Kong Institute for Advanced Study, City University of Hong Kong, Kowloon, Hong Kong 999077, China*

[c]*City University of Hong Kong Shenzhen Research Institute, Shenzhen, Guangdong 518057, China*

[d]*Department of Quantum Matter Physics, University of Geneva, 24 Quai Ernest-Ansermet, 1211 Geneva, Switzerland*

[e]*Department of Physics, University of Warwick, Coventry, CV4 7AL, United Kingdom*

[f]*Electron Spectrometry and Microscopy Laboratory (LSME), Institute of Physics (IPHYS), École Polytechnique Fédérale de Lausanne (EPFL), 1015 Lausanne, Switzerland*

[g]*Catalan Institute of Nanoscience and Nanotechnology (ICN2), Campus UAB, Bellaterra, Barcelona, 08193, Catalonia, Spain*

[h]*Diamond Light Source, Harwell Campus, Didcot OX11 0DE, United Kingdom*

[i]*H.H. Wills Physics Laboratory, University of Bristol, Bristol BS8 1TL, United Kingdom*

[j]*Department of Physics, University of Florida, Gainesville, Florida 32611, USA*

[k]*Department of Computer Science, University of Florida, Gainesville, Florida 32611, USA*

[l]*The Molecular Foundry, Lawrence Berkeley National Laboratory, Berkeley, California 94720, USA*

*Email: danfeng.li@cityu.edu.hk, zhenpingwu@bupt.edu.cn, chunjing@phys.ufl.edu, kejin.zhou@diamond.ac.uk.

[§] *Z. D and M. H contributed equally to this work.*

Content:

Supplementary Information Text:

Growth of precursor films

Reduction processes

X-ray diffraction and atomic force microscopy

Scanning transmission electron microscopy

Transport measurements

X-rays absorption spectroscopy and resonant inelastic X-rays scattering

Density functional theory calculations

Lattice parameters of infinite layer nickelate determined using STEM

Visualization of oxygen atoms using annular bright field imaging

The strain distribution of infinite layer nickelate

Figures S1 to S7

Table S1

SI References

Growth of precursor films

The pristine $(001)_{pc}$ NNO3 films (thickness equal to approximately 30 unit cells) capped with 5 unit cells of STO were deposited using off-axis radiofrequency magnetron sputtering from stoichiometric ceramic targets. The commercial NGO $(001)_{pc}$ substrate was selected as it offers a good lattice match with NNO3, and it also shares the same oxygen octahedral rotation pattern, minimizing the structural mismatch between film and substrate[1]. Before film deposition, the NGO substrates were annealed at 1150°C in flowing oxygen for 10 hours to obtain an atomically flat surface. Deposition from the ceramic target for NNO3 growth was then carried out in a 0.14 Torr atmosphere with an oxygen:argon ratio of 10:35 and RF power of 50 W[2]. Subsequently, the thin STO capping layer was deposited in a working pressure of 0.18 Torr, oxygen:argon ratio of 20:28 and RF power of 60 W. During the deposition of both materials, the substrate temperature was kept at 550°C, measured by a thermocouple inside the heating block.

Reduction processes

The as-grown precursor films were cut into two pieces with the size of 5×2.5 mm^2. Each piece was wrapped by aluminum foil and vacuum-sealed with the hydride powders (both CaH_2 and NaH of ~ 0.1 g) in a Pyrex glass tube. The films were then annealed at different temperatures in a tube furnace (Thermo Fisher Scientific) for synthesis of the infinite-layer NNO2 phase. The ramping rate was kept to 10 °C min^{-1} irrespective of temperature. After reduction, the sample surface was washed with 2-butanone to remove the residual highly reactive reactant.

X-ray diffraction and atomic force microscopy

XRD 2θ-ω symmetric scans and RSM were performed using a Rigaku Smartlab X-ray diffractometer with an X-ray wavelength λ of 1.5406 Å, equipped with a two-bounce Ge (220) monochromator. The surface morphology of the films was examined using a commercial AFM (Veeco Di-Multimode V) in tapping mode. The AFM tips are from AppNano ACTA.

Scanning transmission electron microscopy

Scanning transmission electron microscopy images were obtained using a double-

corrected Thermofisher TITAN Themis electron microscope located in the Interdisciplinary Centre for Electron Microscopy (CIME) at École Polytechnique Fédérale de Lausanne, equipped with a double-corrector DCOR (CEOS) and a high-brightness field emission gun with monochromator, operated at 300 kV in STEM mode. The specific orthorhombic $[001]_{NGO}$ zone axis was chosen as it is the viewing axis along which the oxygen columns are better projected in the Pbnm lattice. In this way, high quality high-angle annular dark field images (HAADF) and annular bright field (ABF) images were simultaneously acquired using a convergence semi-angle of 20 mrad and low beam currents (30 - 40 pA) to prevent damage and contamination. Linear and non-linear scanning distortions were minimized by acquiring image series composed by 24 iterative 90° rotated HAADF & ABF frames, which were aligned using the SmartAlign plug-in for DigitalMicrograph afterwards[3]. The central position associated to each Nd (A-site) atomic column have been estimated using a 2D Gaussian fitting procedure using the Atomap documentation[4].

The removal of oxygen by topochemical reduction can be visualized by the cross-sectional annular bright-field (ABF) STEM images acquired along the [100] zone axis, as shown in Figure S3. In addition, the relative atomic displacements can be identified, revealing the rotation of the oxygen octahedra with the expected $a^-a^-c^+$ tilt pattern as shown in the overlaid structure. For NNO2, the relative atomic displacements observed in the NNO3 film are now suppressed and all the oxygen columns from the Ni-O plane lay in the same horizontal plane.

Transport measurements

The longitudinal and Hall resistivity was measured with a six-point geometry using Al wire bonded contacts. For Hall measurements, an etched Hall-bar pattern was not adopted as the effects of the etching and patterning processes on superconductivity are still being investigated. The temperature and magnetic field dependent resistance and Hall voltages were measured in a Quantum Design Physical Property Measurement System (PPMS) system equipped with a 9 Tesla magnet. The film thickness used to extract the resistivity values was obtained from the XRD Scherrer fitting.

In a few samples, small drops of resistivity are observed (Figure 3b). The dip in resistivity at low temperatures can be an indication of a weak superconductivity (Figure

S5a). Such a downturn in resistivity appears to resemble the same behavior observed in high-quality undoped $LaNiO_2$[5], and more recently $NdNiO_2$[6] and $PrNiO_2$[7]. This resemblance can be taken as universal evidence of a superconducting state even in the undoped parent phase.

To verify if this is a trace of superconductivity, we measured the magnetoresistance of Sample 1 at various temperatures from the base temperature (2 K) to higher temperatures to examine the magnetic field dependence. In Figure S5b, we can clearly see that, for temperature below ~ 8 K, which is at the beginning of the resistivity downturn (Figure S5a), a dip in symmetrized magnetoresistance data at low fields is present. This resistivity downturn at low temperatures can be suppressed by both the magnetic field and temperature. We define the magnetic field where resistivity peaks as a characteristic field, μ_0H^*, and plot it against measurement temperature, as illustrated in Figure S5c. We obtained a temperature dependent characteristic field that is similar to the behavior of the upper critical field, $\mu_0H_{c,2}$, of the infinite-layer nickelates, with a smaller but comparable strength[8-12]. As a comparison, the same magnetic-field-dependent measurements were performed on a sample absent of the resistivity dip (Sample 2). The data is shown in Figure S5d.

X-rays absorption spectroscopy and resonant inelastic X-rays scattering

XAS and RIXS measurements were performed at Beamline I21, Diamond Light Source, UK. As depicted in Supplementary Fig. 6, the crystallographic a–c (or equivalently b–c) planes of all samples aligned with the scattering plane, which was defined by the incident and outgoing beams. r.l.u. are defined (where 2π/a = 2π/b = 2π/c = 1) as $Q = Ha^* + Kb^* + Lc^*$. In the measurements, the spectrometer arm was positioned at a fixed angle $\Omega = 154^{\circ}$, so as to align all samples such that $K = 0$.

XAS spectra were measured at 20 K with the exit slit opening to 50 μm at the Ni L_3 edge. The grazing incidence angle of $\theta_0 = 20^{\circ}$ was configured to probe both the in- and out-of-plane unoccupied states. The XAS data were recorded in TEY/Fluorescence yield with a photodiode and normalized to incoming beam intensity. Both linear vertical (σ) and horizontal (π) polarisations were used. While σ-polarised light probes only in-plane XAS, that is, $I_{ab} = I_{\sigma}$, the out-of-plane XAS were obtained by a combination of

both σ and π polarisations with $I_c = (I_\pi - I_\sigma \sin^2[\theta_0]) / \cos^2[\theta_0]$.

Energy-dependent RIXS measurements were performed at an in-plane position of Q = (−0.35, 0) at a temperature of 20 K, with the exit slit opening to 30 μm corresponding to an average energy resolution of 41 meV (FWHM). The incident energy range was 851.5–854 eV, in steps of 100 meV, to fully capture resonance behaviour across Ni L_3 absorption peaks.

Density functional theory calculations

Density-functional theory calculations for both bulk NNO2 and the heterostructure were conducted using the Generalized Gradient Approximation (GGA) method[13], as implemented in Quantum ESPRESSO. Projector-Augmented Wave (PAW) pseudopotentials[14] were utilized, with the 4*f* electrons treated as core electrons for Nd. In the bulk NNO2 calculations, the self-consistent field (SCF) calculations employed a Monkhorst-Pack grid[15] of 12 × 12 × 12 k-points. For the heterostructure calculations, the SCF calculations were performed with a Monkhorst-Pack grid of 1 × 4 × 4 k-points. For the calculation of the electronic structure of the bulk, although the *a* and *b* in-plane lattice parameters deviate from being exactly the same, breaking R4 symmetry and lowering the symmetry of the Brillouin Zone, the asymmetric effect on the electronic structure is nearly indistinguishable (Figures 5a and 5b in the main text) due to the rather small magnitude.

For investigation of the structural distortion and the corresponding electronic structure at the interface of NNO2/NGO, to circumvent potential complications at the vacuum-material interface, we designed a superlattice that incorporates three layers of NGO as the substrate, connected with five layers of NNO2 in both directions. The structural relaxation is implemented by maintaining the lattice parameters of NGO fixed and relaxing the atomic coordinates of NNO2. The NGO structure is characterized by large out-of-plane octahedral tilts and oxygen distortions in alternating octahedra.

Lattice parameters of infinite layer nickelate determined using STEM

To determine lattice parameters of the $NdNiO_2$ (NNO2) film in Figure. 2f, we identify first the central position of every A-site atomic column by using a 2D-Gaussian fit with Atomap[4]. From these positions, we can then retrieve the in-plane/out-of-plane

parameter of each unit cell of the imaged lattice by measuring the relative horizontal/vertical spacing between two neighboring A-site columns. By doing the average among all the unit cells belonging to the same atomic monolayer, we can estimate the vertical evolution of both structural parameters.

Visualization of oxygen atoms using annular bright field imaging

For the $NdNiO_3$ (NNO3) film (in Figure S3a), the oxygen columns are seen in both Nd-O and Ni-O planes with comparable contrast, which is better illustrated in the intensity profile (Figure S3c); the red and black lines representing the Nd-O and Ni-O columns, respectively, present similar intensity in the oxygen positions. For the NNO2 film on the other hand, upon the removal of the apical oxygen ions in the Nd planes, a clear brightness difference is identified when comparing the oxygen columns linked to the apical (Nd-O) and planar (Ni-O) positions.

The strain distribution of infinite layer nickelate

The drastic reduction in the out-of-plane lattice parameter of the NNO2 film compared to the parent perovskite phase can also be identified when using the geometrical phase analysis (GPA) method, as shown in Figure S4. The color map (ε_{yy}) corresponds to the local variations of the out-of-plane lattice parameter across the image, which is calculated from the relative vertical displacements of the (001) FFT spot with respect to its reference position (chosen to be the $NdGaO_3$ (NGO) substrate). The color of the map adopts negative values (blue-green color) in the NNO2 film, which relates to a reduction of the out-of-plane lattice parameter compared to the substrate. The measured average value is around 3.38 Å, which agrees well with the values obtained from the coordinates of the A-site sublattice.

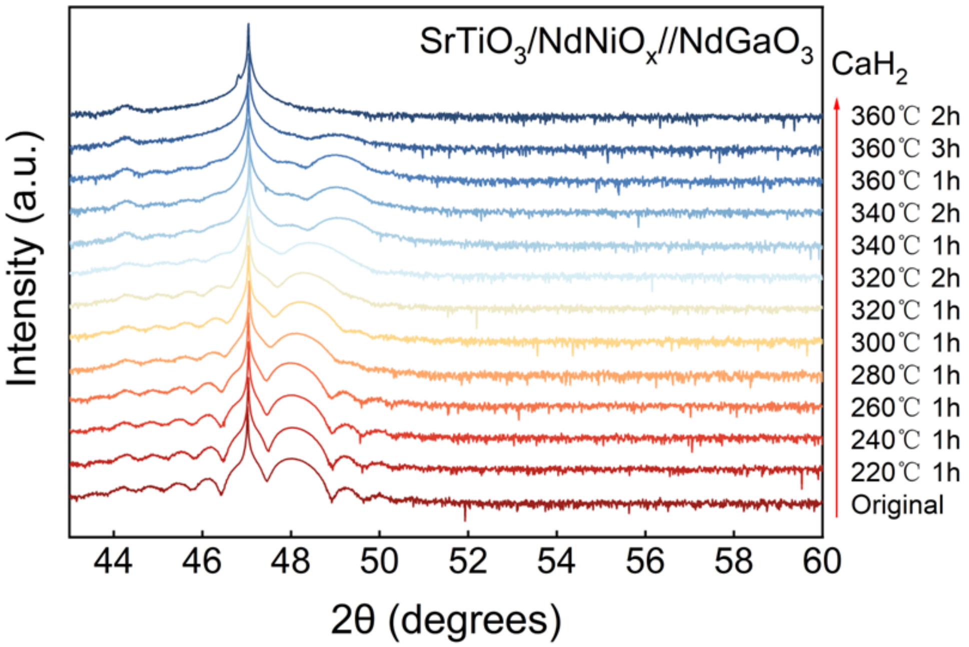

Figure S1. Full reduction process using CaH_2. Limited-range X-ray diffraction θ-2θ scans of the $NdNiO_y$ film on the $NdGaO_3$ substrate after a series of reduction steps. The reactant is CaH_2.

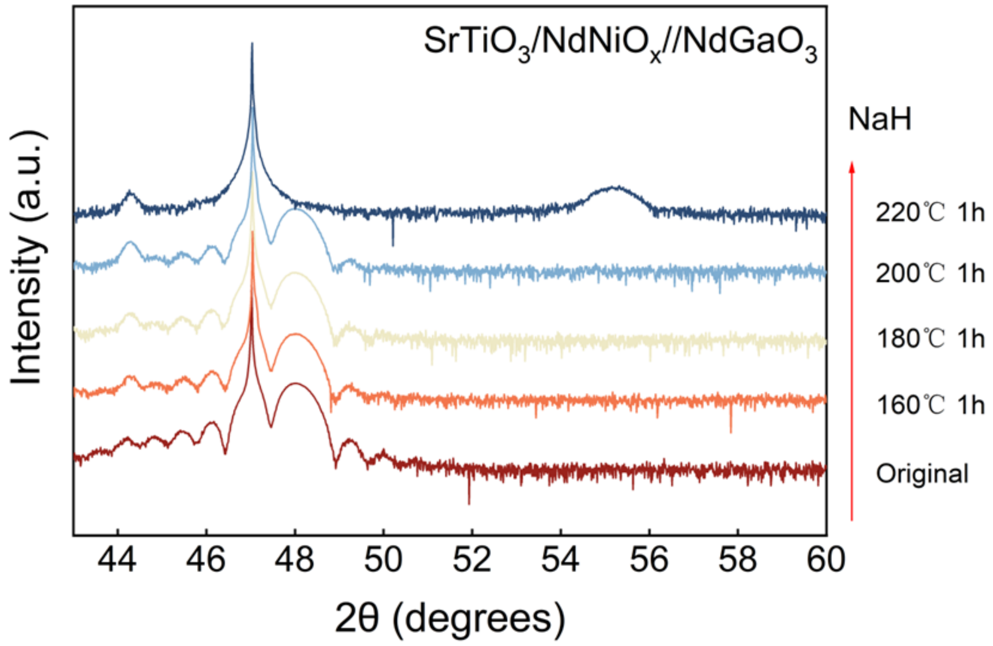

Figure S2. Full reduction process using NaH. Limited-range X-ray diffraction θ-2θ scans of the $NdNiO_y$ film on the $NdGaO_3$ substrate after a series of reduction steps enabled by NaH.

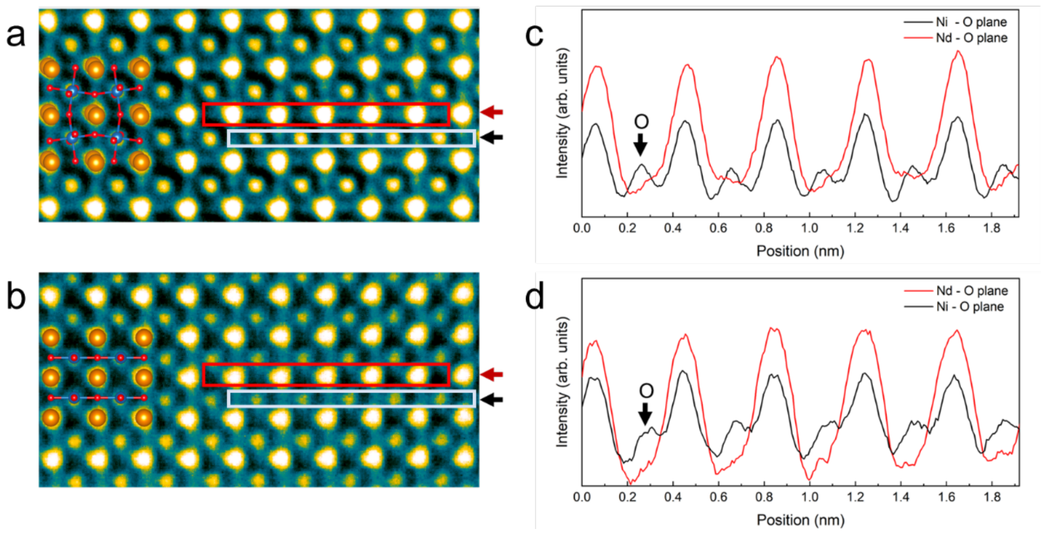

Figure S3. ABF-STEM image of (a) the $NdNiO_3$ film and (b) the $NdNiO_2$ film. The red boxes crop the Nd-O plane, and the cyan boxes crop the Ni-O plane. (c, d) The intensity of ABF-STEM signals along the boxes defined in (a) and (b), respectively.

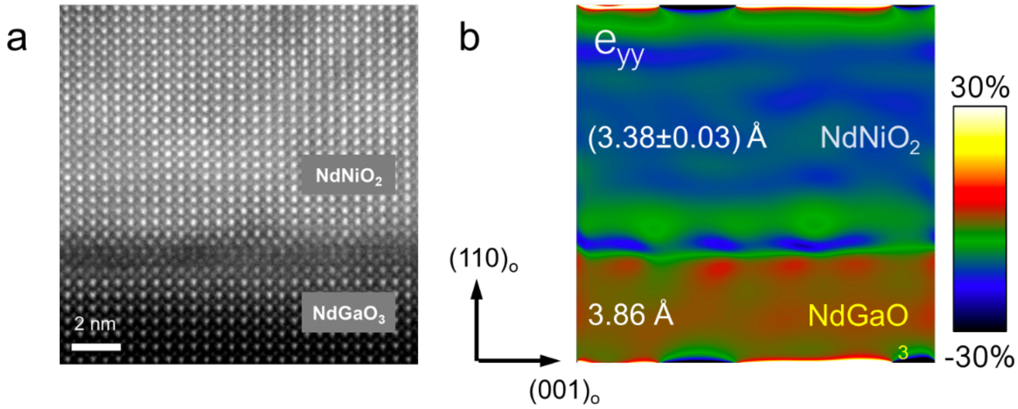

Figure S4. (a) The enlarged HADDF-STEM image of the interface region between the infinite-layer phase and the $NdGaO_3$ substrate. (b) Strain distribution along orthorhombic [001] direction of the same region in (a).

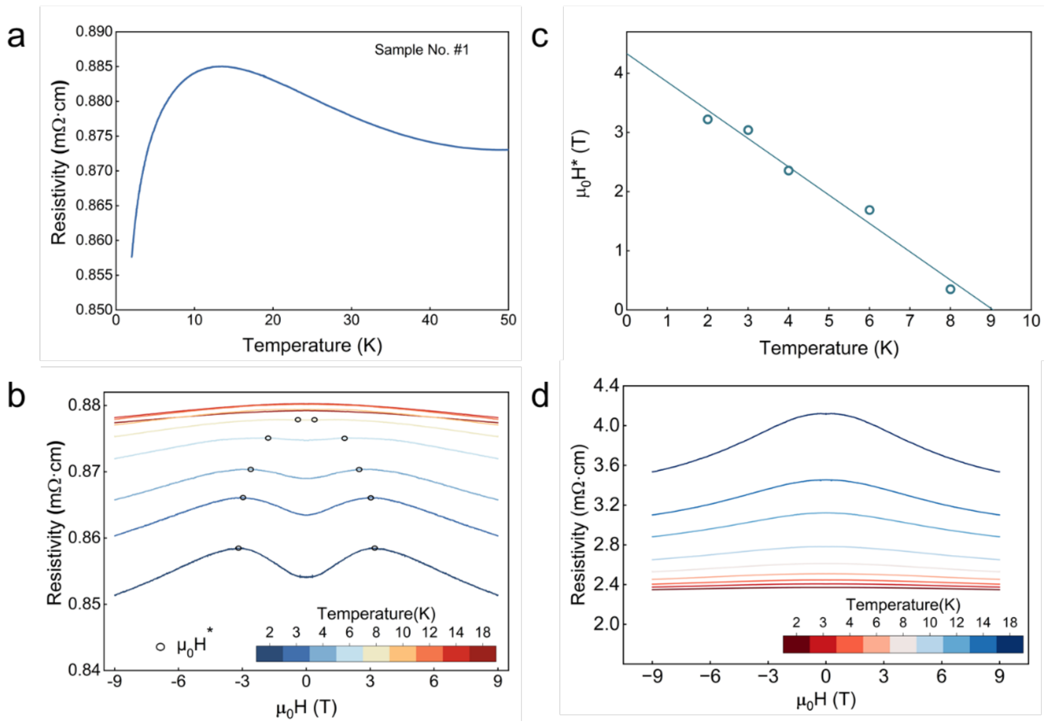

Figure S5. (a) Resistivity versus temperature curves of Sample 1 from 2 K to 50 K. (b) The magnetic-field dependence of resistivity of Sample 1 shows a dip in resistivity. (c) The characteristic field μ_0H^* as a function of temperature. μ_0H^* is defined as the magnetic field values at which the resistivity derivative is minimal. (d) Magnetoresistance of Sample 2 without low-temperature resistivity downturn measured at different temperatures.

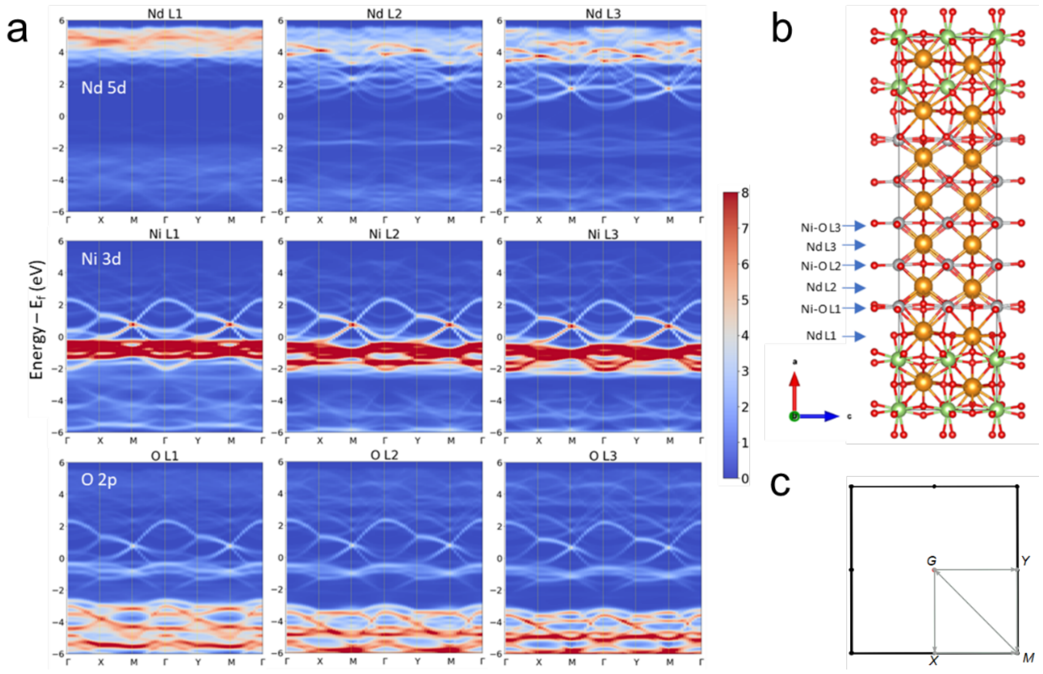

Figure S6. (a) DFT calculated band structure for the heterostructure across different layers, with the heterostructure and each layer shown in (b). The first Brillouin zone and high symmetry points are shown in (c).

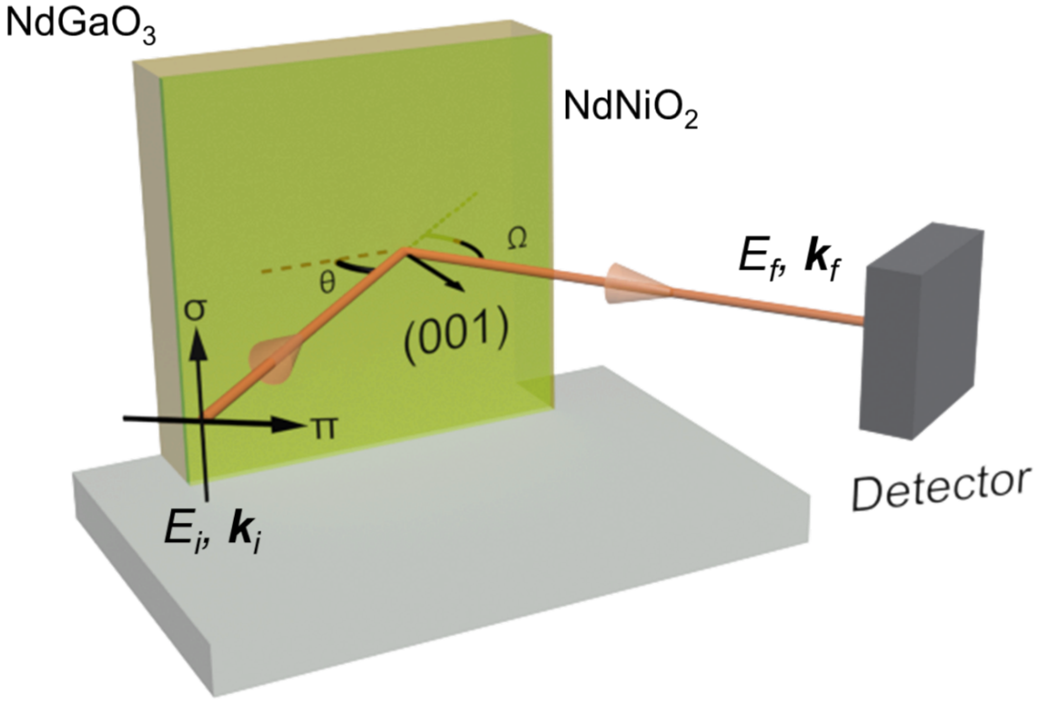

Figure S7. The experimental geometry used for the XAS and RIXS measurements.

Table S1. XRD data, fitting thickness, and resistivity of various NNO2 samples.

**Sample**	**2theta (°)**	**Lattice parameter (Å)**	**Thickness (nm)**	**Resistivity (mΩ·cm) at 300 K**	**Resistivity (mΩ·cm) at 2 K**
1	55.339	3.318	9.67	1.169	0.858
2	55.339	3.318	9.67	1.316	1.949
3	55.430	3.313	10.07	2.050	1.997
4	55.344	3.317	8.83	1.175	1.281
5	55.344	3.317	8.83	1.539	1.792
6	55.149	3.328	9.65	1.503	1.944
7	55.259	3.322	10.35	1.688	1.962
8	55.320	3.319	9.87	1.946	2.816
9	55.358	3.317	10.40	2.121	2.910
10	55.110	3.330	9.48	2.140	3.494

Reference:

(1) García-Muñoz, J. L.; Rodríguez-Carvajal, J.; Lacorre, P.; Torrance, J. B. Neutron-diffraction study of $RNiO_3$ (R=La,Pr,Nd,Sm): Electronically induced structural changes across the metal-insulator transition. *Phys. Rev. B* **1992,** *46*, 4414-4425.

(2) Hadjimichael, M.; Mundet, B.; Domínguez, C.; Waelchli, A.; De Luca, G.; Spring, J.; Jöhr, S.; McKeown Walker, S.; Piamonteze, C.; Alexander, D. T. L., et al. Competition between Carrier Injection and Structural Distortions in Electron-Doped Perovskite Nickelate Thin Films. *Adv. Electron. Mater.* **2023,** *9*, 2201182.

(3) Jones, L.; Yang, H.; Pennycook, T. J.; Marshall, M. S. J.; Van Aert, S.; Browning, N. D.; Castell, M. R.; Nellist, P. D. Smart Align—a new tool for robust non-rigid registration of scanning microscope data. *Adv. Struct. Chem. Imaging* **2015,** *1*, 8.

(4) Nord, M.; Vullum, P. E.; MacLaren, I.; Tybell, T.; Holmestad, R. Atomap: a new software tool for the automated analysis of atomic resolution images using two-dimensional Gaussian fitting. *Adv. Struct. Chem. Imaging* **2017,** *3*, 9.

(5) Osada, M.; Wang, B. Y.; Goodge, B. H.; Harvey, S. P.; Lee, K.; Li, D.; Kourkoutis, L. F.; Hwang, H. Y. Nickelate superconductivity without rare-earth magnetism: $(La,Sr)NiO_2$. *Adv. Mater.* **2021,** *33*, 2104083.

(6) Parzyck, C.; Wu, Y.; Bhatt, L.; Kang, M.; Arthur, Z.; Pedersen, T.; Sutarto, R.; Fan, S.; Pelliciari, J.; Bisogni, V., et al. Superconductivity in the parent infinite-layer nickelate $NdNiO_2$. *arXiv:2410.02007.* DOI: 10.48550/arXiv.2410.02007 (accessed 2024.10.02).

(7) Sahib, H.; Rosa, F.; Raji, A.; Merzoni, G.; Ghiringhelli, G.; Salluzzo, M.; Gloter, A.; Viart, N.; Preziosi, D. Superconductivity in $PrNiO_2$ infinite-layer nickelates. *arXiv:2410.16147.* DOI: 10.48550/arXiv.2410.16147 (accessed 2024.10.21).

(8) Wang, B. Y.; Li, D.; Goodge, B. H.; Lee, K.; Osada, M.; Harvey, S. P.; Kourkoutis, L. F.; Beasley, M. R.; Hwang, H. Y. Isotropic Pauli-limited superconductivity in the infinite-layer nickelate $Nd_{0.775}Sr_{0.225}NiO_2$. *Nat. Phys.* **2021,** *17*, 473-477.

(9) Chow, L.; Yip, K.; Pierre, M.; Zeng, S.; Zhang, Z.; Heil, T.; Deuschle, J.; Nandi, P.; Sudheesh, S.; Lim, Z., et al. Pauli-limit violation in lanthanide infinite-layer nickelate superconductors. *arXiv:2204.12606.* DOI: 10.48550/arXiv.2204.12606 (accessed 2022.04.26).

(10) Wang, B. Y.; Wang, T. C.; Hsu, Y.-T.; Osada, M.; Lee, K.; Jia, C.; Duffy, C.; Li, D.;

Fowlie, J.; Beasley, M. R., et al. Effects of rare-earth magnetism on the superconducting upper critical field in infinite-layer nickelates. *Sci. Adv.* **2023,** *9*, eadf6655.
(11) Ji, H.; Liu, Y.; Li, Y.; Ding, X.; Xie, Z.; Ji, C.; Qi, S.; Gao, X.; Xu, M.; Gao, P., et al. Rotational symmetry breaking in superconducting nickelate $Nd_{0.8}Sr_{0.2}NiO_2$ films. *Nat. Commun.* **2023,** *14*, 7155.
(12) Sun, W.; Li, Y.; Liu, R.; Yang, J.; Li, J.; Wei, W.; Jin, G.; Yan, S.; Sun, H.; Guo, W., et al. Evidence for Anisotropic Superconductivity Beyond Pauli Limit in Infinite-Layer Lanthanum Nickelates. *Adv. Mater.* **2023,** *35*, 2303400.
(13) Perdew, J. P.; Burke, K.; Ernzerhof, M. Generalized Gradient Approximation Made Simple. *Phys. Rev. Lett.* **1996,** *77*, 3865-3868.
(14) Blöchl, P. E. Projector augmented-wave method. *Phys. Rev. B* **1994,** *50*, 17953-17979.
(15) Monkhorst, H. J.; Pack, J. D. Special points for Brillouin-zone integrations. *Phys. Rev. B* **1976,** *13*, 5188-5192.
